# Nutritional Quality and Environmental Impact of Public School Meals: Evaluation of Current Meals and Potential Benefits of Vegetarian Diets for Sustainable Improvement

**DOI:** 10.3390/nu18142269

**Published:** 2026-07-11

**Authors:** Julia Serejo Mello, Ana Clara Rocha Rodrigues, Eduardo Yoshio Nakano, Gabriella Carvalho Medeiros Carvalho Branco, Maria Clara Corrêa de Alcantara, Shila Minari Hargreaves

**Affiliations:** 1 Graduate Program in Health Sciences, Escola Superior de Ciências da Saúde, Universidade do Distrito Federal Professor Jorge Amaury Maia Nunes, Brasília 71503-502, DF, Brazil; juliaserejo22@gmail.com (J.S.M.); mariaclaralcantara.nutri@gmail.com (M.C.C.d.A.); 2 Faculdade de Ciências da Educação e Saúde (FACES), Centro Universitário de Brasília, Brasília 70790-075, DF, Brazil; anaclarapsrocha@gmail.com (A.C.R.R.); gabriella.cmcbranco@gmail.com (G.C.M.C.B.); 3 Department of Statistics, Universidade de Brasília, Brasília 70910-900, DF, Brazil; eynakano@gmail.com

**Keywords:** school feeding, school meals, sustainable diets, environmental impact, National School Feeding Program, nutrition policy, nutritional status, children’s health

## Abstract

**Background/Objectives:** School feeding is a fundamental component of public policies aimed at promoting health, improving educational outcomes, reducing inequalities, and guaranteeing the human right to adequate food. This study aimed to evaluate the nutritional quality of school meals offered to public school students in a federal unit of Brazil, quantify the environmental impacts using carbon and water footprints, and simulate potential reductions through a strict vegetarian menu. **Methods:** This cross-sectional, descriptive study analyzed 130 daily menus (390 meals) from full-time public schools in the Federal District of Brazil in 2024. Nutritional quality was assessed based on energy, nutrients, food groups, degree of processing, and food origin. Carbon and water footprints were estimated using literature-based indicators. A nutritionally adequate strict vegetarian menu was then developed and compared with the observed menus. **Results:** The current menus presented good overall nutritional quality, with high food diversity and predominance of fresh or minimally processed foods. Most nutritional parameters met the recommended levels; however, protein and saturated fat exceeded the recommended limits. Animal-based foods accounted for most of the carbon and water footprints. The simulated strict vegetarian menu demonstrated significantly lower environmental impacts while maintaining nutritional adequacy. **Conclusions:** These findings highlight the importance of integrating nutritional and environmental strategies, such as a weekly “Meatless Monday” initiative alongside food and nutrition education, to improve student health outcomes and reduce the environmental burden of public school meals. Incorporating environmental sustainability criteria into school meal planning and public food procurement may advance nutritional quality, resource efficiency, and climate goals, positioning school feeding programs as strategic instruments for sustainable development.

## 1. Introduction

School feeding programs play a central role in the overall development of children and adolescents and are internationally recognized as important public policies for promoting health, food security, and social equity, particularly in low- and middle-income countries. Adequate school meals are associated with improved nutritional status, cognitive performance, school attendance, and educational outcomes among children and adolescents, fostering healthy dietary habits that can persist into adulthood [1]. These programs are especially important in contexts of socioeconomic vulnerability, where school meals may be the primary source of balanced nutrition throughout the day, positioning school feeding as both an educational and a public health strategy [1].

In Brazil, school feeding is a constitutional right implemented through the National School Feeding Program (PNAE), established in 1955 and strengthened by the 1988 Constitution as a mechanism to guarantee access to adequate food [2]. The program is internationally recognized as one of the largest and most innovative school feeding initiatives worldwide [3]. It aims to ensure access to healthy meals that contribute to growth, development, learning, and the formation of lasting healthy eating habits [4].

The PNAE establishes clear nutritional and operational guidelines, including macronutrient distribution targets; limits on saturated fat, added sugars, and sodium; and a requirement to prioritize fresh or minimally processed foods while restricting ultra-processed products [5]. The program also emphasizes cultural appropriateness, respect for regional food practices, and the promotion of sustainable development by including products from family farming and regional biodiversity [2]. Nutritionists are responsible for planning, executing, and evaluating menus, thereby reinforcing the importance of nutritional quality as a central component of the program [6].

In the Federal District, the program is implemented by the State Department of Education and operates at scale, serving hundreds of schools and more than 400,000 students daily, distributing millions of meals and substantial volumes of food annually [4,7]. This scale highlights not only the program’s nutritional and social importance but also its economic and environmental implications [4].

The dietary recommendations adopted by the PNAE align with international guidelines from the World Health Organization (WHO), which promote dietary patterns based on fruits, vegetables, and minimally processed foods while limiting free sugars, sodium, and saturated fats [8]. These principles are also reflected in the Food Guide for the Brazilian Population, an official dietary guideline published by the Ministry of Health to promote healthy eating and support public food and nutrition policies, including the PNAE. The Guide is internationally recognized for integrating the nutritional, environmental, social, cultural, and economic dimensions of food systems, thereby making sustainability a core element of healthy eating [9].

Food systems play a central role in environmental sustainability, as they are major contributors to global greenhouse gas emissions, intensive freshwater use, soil degradation, and biodiversity loss [10]. Furthermore, food production is directly associated with deforestation, biodiversity loss, soil degradation, and increasing pressure on natural resources [11]. Animal-based foods, in particular, are associated with higher environmental impacts due to livestock production, which involves high levels of water consumption, land use, and greenhouse gas emissions, including carbon dioxide, methane, and nitrous oxide [12]. In this context, school feeding programs represent strategic opportunities to promote sustainable food systems, as menu planning and public procurement policies can influence large-scale consumption patterns and encourage more sustainable dietary patterns [3].

The need to integrate nutritional and environmental dimensions is reinforced by the concept of the Global Syndemic of obesity, undernutrition, and climate change, which highlights the interconnected nature of these challenges and the importance of comprehensive policy responses [13]. Scientific evidence also demonstrates that plant-based diets can simultaneously improve public health outcomes and reduce environmental impacts [3,14]. These findings suggest that optimizing school menus by prioritizing plant-based foods and reducing reliance on high-impact animal-based products can contribute to climate change mitigation and reinforce schools’ educational role in promoting sustainable habits [15].

International research has increasingly integrated assessments of the nutritional quality of school meals with analyses of their environmental impacts. Dahmani et al. (2024) demonstrated that expanding the availability of vegetarian meals in European schools can reduce greenhouse gas emissions without compromising nutritional adequacy [16]. In France, researchers modeled scenarios to reduce the environmental footprint of school meals while preserving nutritional adequacy. Their results showed that increasing vegetarian meal offerings and reducing ruminant meat consumption could substantially reduce environmental impacts while maintaining nutritional quality [17].

A study conducted in an Italian school demonstrated that increasing the frequency of legumes, reducing the provision of white and red meat, and eliminating processed meat from school menus resulted in substantial reductions in both carbon and water footprints [18]. In Brazil, initiatives such as “Meatless Monday” (“Segunda Sem Carne”) in public schools in São Paulo have demonstrated significant environmental benefits [19]. The movement also has an important educational role by encouraging critical reflection on food choices and sustainability [20].

Although evidence on sustainable diets is growing, studies that simultaneously assess nutritional quality, degree of food processing, and environmental footprints within large-scale public school feeding programs remain limited. In the Brazilian context, most research has focused on the nutritional evaluation of school menus or the presence of ultra-processed foods, with fewer studies integrating these different dimensions into a comprehensive framework [21,22,23].

This study addresses an important gap in the literature by integrating nutritional quality, degree of food processing, food origin, and environmental footprints into the assessment of school meals within one of the world’s largest public school feeding programs. In addition, it simulates a nutritionally adequate strict vegetarian menu, including quantitative projections of environmental impact reductions. The findings provide relevant evidence to support the development of sustainable food policies and to strengthen the debate on strategies such as “Meatless Monday,” reducing ultra-processed foods, and promoting healthier, more sustainable food systems.

Accordingly, this study aimed to assess the nutritional quality of school meals in the Federal District in relation to national PNAE guidelines, analyze their environmental impacts, and identify strategies to improve sustainability through menu modifications, including the development of a strict vegetarian menu proposal.

## 2. Materials and Methods

This study was conducted in two complementary stages. The first stage consisted of a descriptive, cross-sectional, quantitative analysis of school meal menus, while the second stage involved developing a strict vegetarian menu proposal with lower environmental impact. The study was based on the analysis of menus from public schools in the Federal District, Brazil, covering elementary school II (ages 11 to 14) and/or high school (ages 15 to 17) on a full-time basis, where students receive three meals per day designed to provide at least 70% of their daily nutritional requirements, as established by national legislation [4]. The same menu is followed at both educational levels, with no differences in meal composition between age groups.

Menus were obtained from official public-domain documents available on the State Department of Education’s website. Because the study relied exclusively on publicly available aggregated data and did not involve direct human participation, ethical approval by an Institutional Review Board was not required [24]. Menus are developed according to regional planning criteria that consider cultural and logistical characteristics of different areas of the Federal District. Schools are grouped into geographic service lots, with all schools within the same lot receiving the same menu cycle.

The PNAE establishes national nutritional standards for school menu planning across Brazil, including macronutrient distribution targets and specific limits: protein between 10% and 15% of total energy intake, carbohydrates between 55% and 65%, total fat between 15% and 30%, saturated fat limited to 7%, added sugars up to 7%, and sodium up to 1400 mg. Additionally, menus must include heme iron sources at least four times per week [4]. In full-time schools, the daily menu is served in three meals throughout the school day: a morning meal (10:00 to 10:30), lunch (11:45 to 12:30), and an afternoon meal (14:00 to 14:30). The program is financed through public funding provided by federal and local authorities [4].

Menus are organized on a bimonthly basis, taking into account seasonality, procurement contracts, and regional characteristics. Food items tend to repeat within each bimester due to procurement cycles and logistical considerations. The school year is divided into six bimesters, with two to nine weekly menu variations offered per bimester in 2024. Across all six bimesters, 26 distinct weekly menu variations were identified. One representative week was selected from each variation, resulting in a sample of 26 weekly menus. Since each week consists of five school days and three meals per day, the final sample comprised 130 daily menus and 390 meals [24,25]. In cases where the first week included holidays or incomplete information, the following week was used instead. This sampling strategy was based on the observation that menus vary within the week but tend to repeat throughout the bimester.

Nutritional analysis was conducted using standardized per capita values provided by the school feeding management body, adjusted by age group. The analysis included total energy (kcal), carbohydrates, proteins, total fats, saturated fats, dietary fiber, added sugars, and sodium, expressed both in absolute values and as percentages of total energy intake. Data were obtained from the Brazilian Food Composition Table (TACO) and the Brazilian Food Composition Table of the University of São Paulo (TBCA). For industrialized foods, nutritional information from product labels was used, considering the brands acquired during the study period [26,27].

Foods were classified according to three criteria: (i) food groups, based on the Food Guide for the Brazilian Population: beans, grains, roots and tubers, vegetables, fruits, nuts, milk and cheese, and meat and eggs (including fish); (ii) degree of processing, using the NOVA classification: fresh or minimally processed, processed, ultra-processed, and culinary ingredients; and (iii) origin: animal or plant-based [9]. Foods containing both plant- and animal-derived ingredients were classified as plant-based when the majority of their components were of plant origin (e.g., bread and pasta) and as animal-based when predominantly composed of animal-derived ingredients, even if containing plant-based components (e.g., tuna with vegetable broth).

Seasonings and preparation details were excluded from the analysis due to insufficient description in the menus and their minimal contribution to nutritional and environmental outcomes. To standardize calculations, foods were analyzed in their raw form, and standard per capita values for salt and oil were applied in accordance with national recommendations.

The environmental impact was assessed by estimating the carbon and water footprints of the foods included in the school menus. The calculations were based on reference values reported in the literature, obtained from the document “Footprints of Food and Culinary Preparations Consumed in Brazil” [28], which provides carbon footprint (kg CO_2_ eq) and water footprint (L) values per 100 g of food or culinary preparation. These values were converted proportionally to the serving sizes offered in the school menus. The carbon and water footprints of all foods served in each meal were then summed to obtain the total environmental impact of the meal and subsequently aggregated to estimate the impact of the daily menu. All calculations were performed in Microsoft Excel 365 (Microsoft Corporation, Redmond, WA, USA). Descriptive statistics, including means, standard deviations (SD), and percentages, were used to summarize the results. The analytical unit adopted in the study was the daily menu, corresponding to the three meals served on a school day. When specific foods were not included in the database, such as tilapia filet, alternative sources were used [29]. Adjustments were made for specific cases, such as converting powdered milk values to ensure comparability.

All menu data were entered into a standardized Microsoft Excel 365 spreadsheet specifically developed for this study. Each food item listed in the menus was recorded with its corresponding per capita serving size and linked to nutritional composition, carbon footprint, and water footprint values obtained from reference databases. To facilitate analysis, foods were coded and grouped according to three predefined classification systems: food groups based on the Brazilian Dietary Guidelines, NOVA food processing categories, and origin (plant- or animal-based). Nutritional and environmental indicators were calculated at the food-item level and subsequently aggregated by meal and by daily menu. The spreadsheet structure enabled automatic calculation of energy, macronutrients, dietary fiber, added sugars, sodium, carbon footprint, and water footprint through embedded formulas. Summary tables were then generated to estimate mean values, standard deviations, percentages, and the relative contribution of each food group and classification category to the overall nutritional profile and environmental impact of the menus.

In the second stage, a strict vegetarian menu was developed to simulate a potential intervention. The proposed menu excluded all animal-based food groups and was designed in accordance with the Food Guide for the Brazilian Population [9], ensuring nutritional adequacy through combinations of plant-based food groups. Additional foods not currently included in procurement lists, such as tofu, textured soy protein, lentils, and chickpeas, were incorporated to improve feasibility and diversity. Nutritional composition and environmental impacts of the proposed menu were calculated using the same methods as those used for the observed menus. A weekly menu projection was also developed to illustrate practical implementation possibilities.

Data was presented using descriptive statistics, including means, standard deviations, and percentages. Daily menus (three meals) were considered the unit of analysis. Nutritional values were compared with national recommendations, and environmental indicators were compared between observed and simulated menus. Data was organized and analyzed using Microsoft Excel 365.

## 3. Results

The final sample consisted of 26 weekly menus, corresponding to 130 daily menus and a total of 390 meals analyzed. Overall, in 2024, 910 daily menus were made available through the School Feeding Program of the Federal District, and the analyzed sample accounted for 14.28% of this total.

Regarding nutritional composition, the analyzed menus had a mean energy value of approximately 1190 kcal per day, corresponding to the expected minimum energy supply for full-time students, whose meals are designed to cover at least 70% of daily nutritional requirements [4]. Table 1 presents the nutritional profile of the analyzed school meals. The mean macronutrient distribution was 59% carbohydrates, 22% total fats, and 19% proteins. When compared with the parameters established by national guidelines, most nutrients met recommended levels, including total energy, carbohydrates, total fats, dietary fiber, added sugars, and sodium. However, the mean protein content exceeded the recommended upper limit, and saturated fat intake exceeded the maximum recommended threshold of 7% of total energy intake [4].

A high level of food diversity was observed across the menus, with 68 distinct food items identified. Among these, 54 items (79.4%) were classified as fresh or minimally processed foods, six items (8.8%) as culinary ingredients, four items (5.9%) as processed foods, and four items (5.9%) as ultra-processed foods.

**Figure 1:**
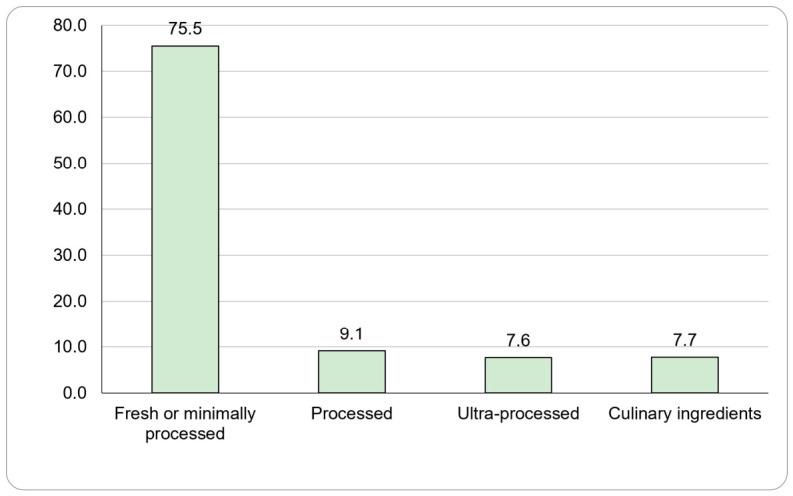
Caloric contribution of each food group, in percentage, according to the NOVA classification, in the 130 menus analyzed.

The proportional caloric contribution of foods according to the NOVA classification showed that the majority of energy intake came from fresh or minimally processed foods, with smaller contributions from processed and ultra-processed foods. This distribution reflects adherence to dietary guidelines that emphasize the consumption of minimally processed foods (Figure 1) [9].

**Figure 2:**
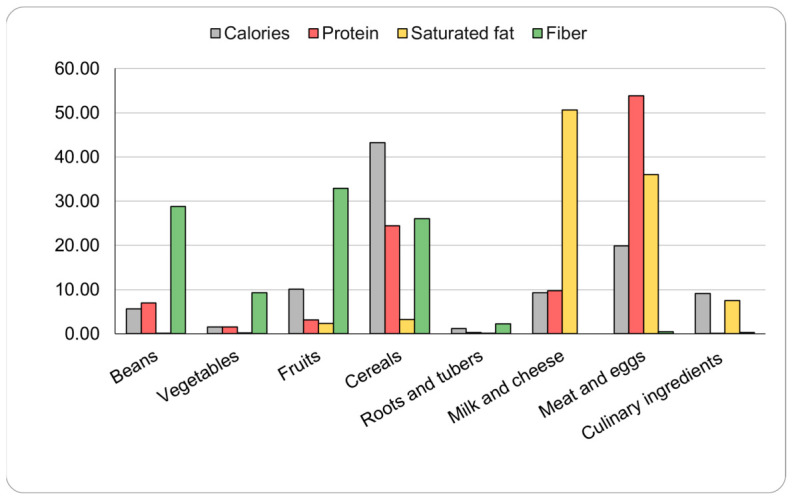
Percentage distribution of nutrients in the 130 menus analyzed, by food group according to the Food Guide for the Brazilian Population.

When foods were grouped according to the categories proposed by the Food Guide for the Brazilian Population [9], cereals (grains) were identified as the primary source of energy in the menus (Figure 2). This plant-based group also contributed significantly to carbohydrate intake and dietary fiber. Beans, cereals, and fruits were the main contributors to fiber intake, although the overall fiber supply averaged 13 g per day, corresponding to approximately 11 g per 1000 kcal, which is below the recommended intake of 14 g per 1000 kcal for individuals aged 10 years and older [30].

The meat and eggs group, although not the largest contributor to total energy intake, accounted for the highest protein intake and was one of the main contributors to saturated fat, second only to the milk and cheese group (Figure 2). Culinary ingredients were analyzed separately because they did not fit into other food-group categories, and the nut group was not displayed in the figure because no items were classified into it.

**Figure 3:**
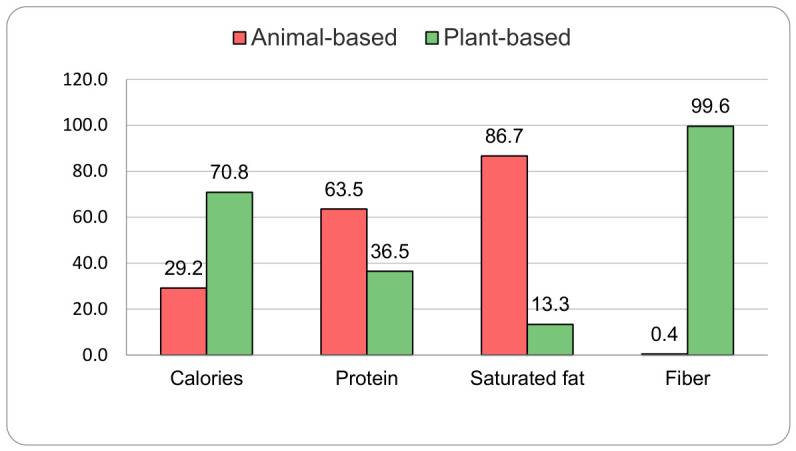
Percentage distribution of nutrients according to food source.

When foods were classified by origin, plant-based foods contributed the majority of total energy intake, whereas foods of animal origin contributed disproportionately to protein and saturated fat intake. In contrast, dietary fiber intake was derived almost exclusively from plant-based foods, reinforcing the importance of these foods in improving dietary quality (Figure 3).

From an environmental perspective, the analysis of the menus showed a mean carbon footprint of 2665.4 g of carbon dioxide equivalent per day, with a standard deviation of 1296.9 g, and a mean water footprint of 3212.8 L per day, with a standard deviation of 1168.6 L. These values represent the estimated greenhouse gas emissions and water consumption associated with producing the foods included in the menus.

**Figure 4:**
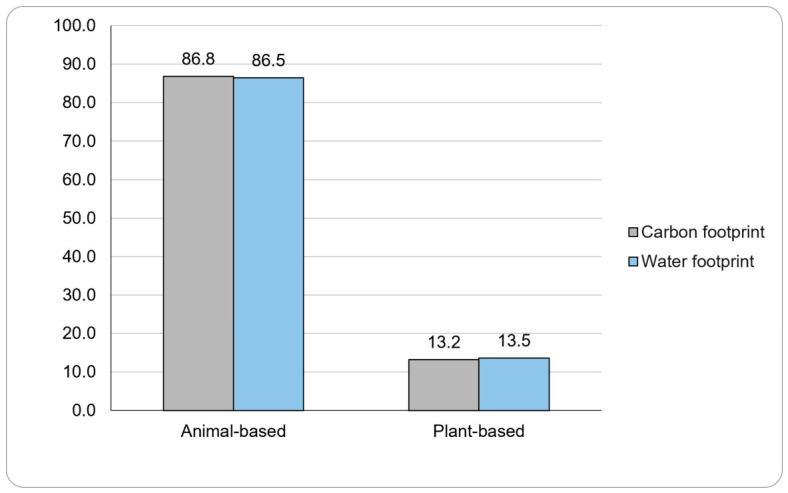
Percentage of environmental footprints of animal- and plant-based foods among the 130 menus evaluated.

When environmental impacts were stratified by food origin, animal-based foods accounted for the largest share of both carbon and water footprints. Although these foods accounted for less than 30% of total energy intake, they were responsible for more than 86% of the menus’ total environmental impact. In contrast, plant-based foods contributed a substantially smaller share to both environmental indicators, demonstrating a lower environmental burden associated with their production and consumption (Figure 4).

**Figure 5:**
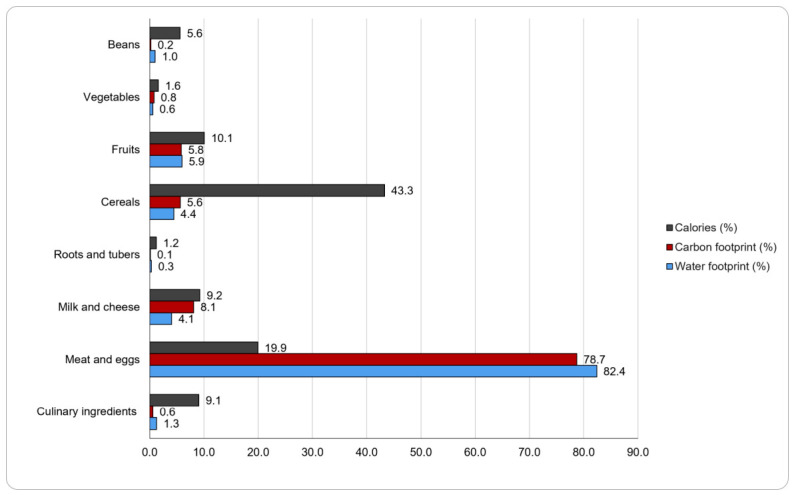
Relative values of the water and carbon footprints and the caloric contribution of each food group among the 130 menus evaluated.

When analyzing environmental impacts by food group, the meat and eggs group was identified as the largest contributor to both carbon and water footprints, despite not being the main contributor to caloric intake. On the other hand, food groups such as grains, fruits, and vegetables exhibited relatively low carbon and water footprints, indicating a lower environmental impact associated with their inclusion in the menus (Figure 5).

Based on these findings, a strict vegetarian menu was developed as an intervention proposal. This menu excluded the meat, eggs, milk, and cheese groups and consisted exclusively of plant-based foods, organized according to the Food Guide for the Brazilian Population [9]. To ensure nutritional adequacy, adjustments were made, including increasing the per capita supply of vegetables and incorporating plant-based protein sources such as legumes and soy-based products. The inclusion of foods such as tofu, textured soy protein, lentils, and chickpeas was suggested to enhance the nutritional quality and feasibility of the proposed menu, although these items are not currently part of the standard procurement list for school feeding in the Federal District (Table 2).

**Figure 6:**
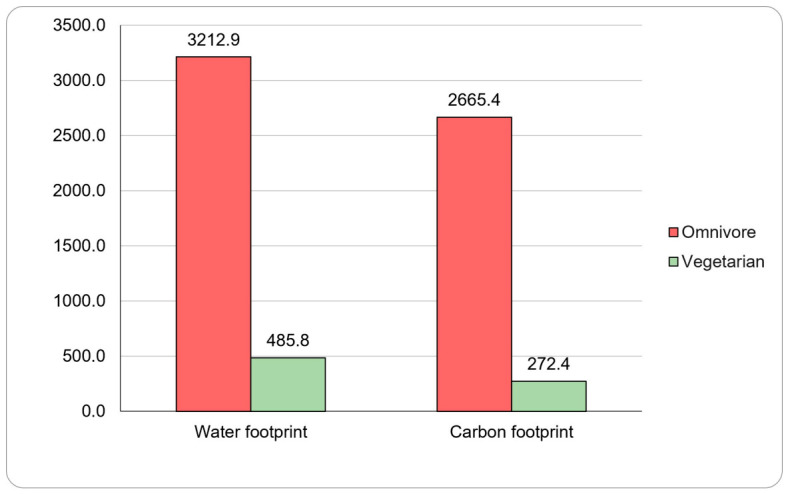
Carbon footprint (eqCO2) and water footprint (liters) of the proposed menu, in comparison with the mean of the 130 menus analyzed.

The comparison between the currently offered menus and the proposed strict vegetarian menu revealed a significant reduction in environmental impacts (Figure 6). The vegetarian menu demonstrated substantially lower carbon and water footprints than the mean values observed across the 130 menus analyzed. Despite these reductions, the proposed menu remained compliant with national nutritional recommendations, ensuring adequate energy intake and macronutrient distribution (Table 3).

**Figure 7:**
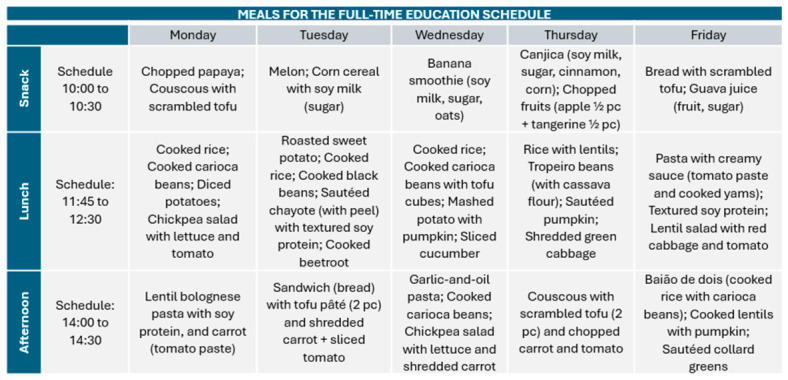
Simulation of a strict vegetarian weekly menu for full-time school meals.

**Figure 8:**
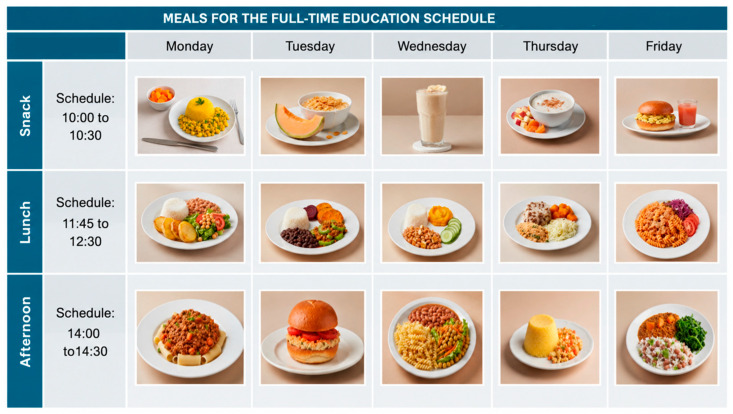
Illustrated simulation of a strict vegetarian weekly menu for full-time school meals.

To further illustrate the feasibility and diversity of the proposed intervention, a weekly vegetarian menu projection was developed, presenting different meal combinations based on plant-based food groups. Both written (Figure 7) and illustrated (Figure 8) versions of this weekly menu were created to support visualization and potential implementation in the school feeding context.

## 4. Discussion

The findings of this study indicate that, overall, school meals provided in the public system of the Federal District in 2024 have high nutritional quality and align with principles established both by the PNAE and the Food Guide for the Brazilian Population [9], which emphasize dietary patterns based on unprocessed and minimally processed foods, promote environmentally sustainable food choices, and recommend avoiding the consumption of ultra-processed foods. The menus analyzed demonstrate a wide variety of foods and are predominantly composed of fresh or minimally processed items, reinforcing the PNAE’s role as a health promotion policy [3]. Despite these positive aspects, the study also identified important environmental challenges. Although animal-based foods account for less than 30% of total energy intake in the menus, they account for more than 86% of the associated water and carbon footprints. This finding suggests that, despite substantial adherence to nutritional recommendations, opportunities remain to further align menu planning with the sustainability principles promoted by the PNAE [5] and the Food Guide for the Brazilian Population [9].

The importance of school meals in improving the nutritional adequacy of children and adolescents has been widely documented in the scientific literature, with studies showing better nutritional quality among children who consume school meals than among those who rely on packed lunches or meals outside school settings [31,32]. A recent review of school meal programs across 29 countries found that most school menus met or exceeded 30% of recommended daily nutrient requirements and were classified as having medium to high nutritional quality. However, the authors also identified important nutritional gaps, particularly regarding fiber intake, as well as substantial variability in menu quality across countries, age groups, and meal types. These findings reinforce that, although school feeding programs play a crucial role in promoting healthy diets, continuous evaluation and improvement of menu composition remain necessary to optimize both nutritional and health outcomes [33].

Among the findings collected in our study, it is noteworthy that fresh and minimally processed foods account for 79.4% of the items offered and 75.5% of the total caloric value. These values fall within a wide range of variability in public school feeding in Brazil. This scenario is comparable to positive examples reported in the literature, such as a study conducted in a municipality in the North region of Brazil, which showed that 50% of public food purchases were allocated to fresh and minimally processed foods [34]. On the other hand, it contrasts with findings that reveal broader challenges, such as the study by Santo et al., which identified a high prevalence of ultra-processed foods in students’ menus in the state school system of a city in the Central-West region of Brazil [21]. Furthermore, the prevalence of ultra-processed foods may be inversely associated with the acquisition of foodstuffs from family farms [35].

Compared with another study conducted in the Federal District in 2021 by Assaf, the present study shows a more favorable scenario, with a greater presence of fresh and minimally processed foods, in accordance with the Food Guide for the Brazilian Population [9,22]. In Assaf’s study, the availability of fruits and vegetables in public school menus was insufficient, remaining below the levels recommended by PNAE guidelines. Although some nutritional aspects met regulatory requirements, overall adequacy in fruit and vegetable provision was limited. Nevertheless, that study highlighted a reduction in the use of processed and ultra-processed products, indicating progress toward improving the nutritional quality of school meals [22].

It is important to note that the analyses conducted in this study were based on school menus from 2024, prior to the implementation of the new PNAE legislation, which establishes maximum limits on the inclusion of ultra-processed foods in school meals [36]. The updated regulation strengthens the emphasis on fresh and minimally processed foods by requiring that a growing share of program resources be allocated to their acquisition. While the 2024 menus already demonstrated a predominance of these food categories, the new regulatory framework further reinforces this priority and underscores the importance of continuous monitoring and refinement of school feeding practices [36].

From a nutritional perspective, this study’s finding that cereals play a dominant role in plant-based caloric and protein intake is consistent with the literature. A recent review highlights that cereals, particularly whole grains, are important sources not only of energy but also of sustainable plant protein and beneficial components such as fiber and phytochemicals [37]. In addition to cereals, other food groups, namely legumes, fruits, and vegetables, provide important sources of fiber, micronutrients, and phytochemicals [26,27]. Recent reviews indicate that dietary fiber intake is inversely associated with mortality from cardiovascular diseases and cancer, and that plant-based foods such as fruits, legumes, and whole grains account for a substantial proportion of total dietary fiber intake [38,39].

In the present study, beans, cereals, and fruits were identified as the main contributors to the fiber content in school meals. Although the PNAE does not establish a specific minimum requirement for fiber, a minimum intake of 14 g per 1000 kcal is recommended for the general population aged 10 years and older. The study found that the average fiber supply of the menus was 13 g, corresponding to approximately 11 g per 1000 kcal, which is below the recommended level. These findings highlight the importance of strengthening the inclusion of fiber-rich foods in school menus [30].

The identification of protein and saturated fat content in school meals also deserves attention. Compared with PNAE recommendations, a slight excess of protein and saturated fat was observed, attributable to the marked presence of foods of animal origin [4]. Supporting this observation, the literature indicates a strong association between high consumption of red meat and full-fat dairy products and increased saturated fat intake [40]. Wambogo et al. reported that in the United States population aged 2 years and older, saturated fat from dairy products accounts for approximately 28% of total intake, followed by meats (22.1%) and, to a lesser extent, plant sources. This pattern supports the finding that meats, eggs, milk, and cheeses are major contributors to saturated fatty acid intake, with potential clinical implications, including increased cardiovascular risk [41].

Although saturated fat may partially contribute to the observed associations, the health implications of animal-source food consumption extend beyond this nutrient alone. Evidence from prospective cohort studies and meta-analyses indicates that higher consumption of red and processed meat is associated with increased risks of all-cause mortality, cardiovascular disease, and type 2 diabetes [42,43,44]. In contrast, vegetarian and pescetarian dietary patterns have been associated with lower risks of overall, colorectal, and pancreatic cancer [45]. These associations are not fully explained by saturated fat intake, as other components of animal-source foods, including heme iron, trimethylamine N-oxide (TMAO), and compounds formed during high-temperature cooking, have been proposed as potential contributors [46]. Furthermore, health outcomes are influenced by overall dietary patterns, highlighting the importance of evaluating foods within the broader context of the diet rather than focusing on individual nutrients alone.

In this context, technical adjustments to school menus, such as diversifying protein sources and prioritizing plant-based preparations, can help reduce saturated fat content without compromising nutritional adequacy. These adjustments align with official guidelines that encourage variety and balance among food groups while also supporting environmental sustainability and addressing the interconnected challenges of obesity, undernutrition, and climate change described by the Global Syndemic framework [4,13].

Unlike previous studies that focused primarily on nutritional adequacy, food processing, or environmental indicators separately, the present study integrated multiple dimensions of menu quality, including nutritional composition, degree of processing, food origin, and environmental footprints. This comprehensive approach provides a broader understanding of the trade-offs and synergies between health promotion and environmental sustainability in school feeding programs.

It is widely accepted that animal-based diets have a greater environmental impact, and our study found precisely that. In the literature, Kustar and Patino-Echeverri (2021) demonstrated that vegan dietary patterns can reduce land use by 50–86%, water consumption by 22–70%, and greenhouse gas emissions by 21–70% compared with omnivorous diets [47]. Similarly, research conducted by Clark et al. (2022) at the University of Oxford, analyzing approximately 57,000 food products in the United Kingdom and Ireland, found that plant-based foods have the lowest environmental impacts, whereas animal-based products such as red meat, fish, and cheeses have among the highest [48].

Studies conducted in school settings reinforce these findings. In the United States, a simulation evaluating school meal item substitution found that replacing cow’s milk with soy milk reduced meal-related greenhouse gas emissions (GHGE) by 22–25%, while replacing beef with plant-based alternatives reduced GHGE by 16–20%. The authors highlighted the potential magnitude of these changes, noting that GHGE associated with school meal programs accounts for approximately 2% of all annual diet-related emissions of the United States [49].

Dahmani et al. (2024) [16] showed that expanding vegetarian meal options in European schools can substantially reduce greenhouse gas emissions while maintaining nutritional adequacy. Their findings support the feasibility of integrating sustainability criteria into school menu planning without compromising the nutritional objectives of school feeding programs [16]. However, Marty et al. (2024) emphasized that the successful implementation of such strategies depends not only on nutritional and environmental performance but also on student acceptance and adherence, highlighting the importance of considering behavioral and cultural factors when proposing menu modifications [50].

Beyond simulation studies, intervention studies conducted in real-world school settings have demonstrated similar results. In a Swedish municipality, a four-week sustainable lunch intervention was implemented in three schools. The revised menus reduced the provision of meat and dairy products while increasing the inclusion of vegetables and root crops. This optimization resulted in a 40% reduction in greenhouse gas emissions without compromising nutritional quality. Importantly, meal satisfaction and food waste did not differ from those observed with the standard menu [51].

In Brazil, Camargo (2023) identified that menus in the São Paulo public school system had a high carbon footprint (46.5 thousand tons of CO_2_ equivalent) and water footprint (660.3 thousand liters), largely due to the extensive use of red meat (356.8 tons in one month) [23]. Taken together, these findings indicate that animal-source foods are the main contributors to the environmental impacts of school meals. Consistent with national and international evidence, foods of animal origin in the present study accounted for more than 86% of the carbon and water footprints of the evaluated menus, despite contributing less than 30% of total energy intake.

Data from the Brazilian Vegetarian Society (SVB, 2022) further support this perspective, indicating that abstaining from animal-based foods for a single day can save approximately 3400 L of water per person, in addition to reducing grain use for animal feed, land occupation, and pollutant emissions. In line with this, strategies such as adopting the “Meatless Monday” campaign have been identified as viable and effective options for reducing environmental impacts without compromising nutritional adequacy [37,38].

Based on the sample analyzed and the number of students served, this study estimated that adopting a strict vegetarian menu in the Federal District’s public school system could result in daily savings of approximately 103 million liters of water, 212 tons of soybeans, 72 hectares of land, and the prevention of 424 tons of greenhouse gas emissions. Annual projections, considering approximately 52 school days with meatless meals, indicate potential savings exceeding 5 billion liters of water, 11 thousand tons of soybeans, and more than 22 thousand tons of emissions [17].

The estimated soybean savings associated with the proposed vegetarian menu reflect the greater efficiency of direct human consumption of plant proteins. Since a substantial proportion of global soybean production is used as animal feed, reducing animal-source foods can decrease overall demand for soy despite the inclusion of soy-based products on menus [52].

The study also simulated the feasibility of implementing a more sustainable menu that meets students’ nutritional needs at the local level. Although the proposal focuses on introducing a strict vegetarian diet once per week, the weekly menu was designed to illustrate a variety of possible meal combinations applicable throughout the school year. The PNAE has increasingly incorporated sustainability into its agenda, and the findings of this study aim to inform the development of strategies aligned with food security, environmental responsibility, and public procurement policies [53].

A balanced school feeding program is fundamental to ensuring adequate physical growth and cognitive development. Regular access to nutritious meals during childhood improves anthropometric outcomes, learning capacity, and school attendance. Evidence also demonstrates that school feeding initiatives are associated with improved dietary quality and enhanced food security among vulnerable populations, reinforcing their role as effective social protection policies [1]. In addition, such programs help reduce inequalities by ensuring equitable access to food and supporting educational participation. The Brazilian school feeding program stands out for its efficient organization, the direct procurement of food from family farmers, and the integration of nutrition education initiatives. As highlighted by Liguori et al. (2024), its success depends on implementation quality and community engagement, areas in which Brazil demonstrates strong performance [54].

Building on these findings, it is essential to recognize that the environmental impacts identified in this study have direct implications for public policy. Although sustainability is embedded within existing frameworks such as the PNAE, the results suggest a gap between guidelines and current practices [4]. Given the disproportionate contribution of animal-based foods to carbon and water footprints, school feeding programs are strategic platforms for aligning policy and practice by integrating environmental criteria into menu planning and procurement. This direction is consistent with global trends toward sustainable diets, as highlighted by the EAT-Lancet Commission [55], which emphasizes the importance of shifting toward more plant-based dietary patterns. In large-scale programs such as the PNAE, such alignment can enhance both nutritional outcomes and environmental sustainability.

Finally, some limitations should be acknowledged. The lack of detailed information on menu preparation, including cooking methods and seasonings, limited the analyses, leading to the use of raw food data to minimize bias. Additionally, menus represent planned meals and may differ from actual implementation due to logistical factors, school events, and variations in student consumption. Limitations related to environmental footprint databases were also identified, including the need to use proxy data for certain foods (e.g., tilapia filet) and potential mismatches between menu items and database entries [28].

Importantly, this study focused on analyzing prescribed diets and simulating menu proposals. Aspects related to implementation, such as financial feasibility, technical feasibility, community acceptance, and student adherence, were beyond its scope and should be addressed in future research. Such investigations are essential to ensure that proposed improvements are both practical and sustainable, thereby strengthening the effectiveness and impact of school feeding programs.

## 5. Conclusions

This study demonstrates that the Federal District School Feeding Program provides nutritionally adequate meals that are largely consistent with the principles of the National School Feeding Program and the Food Guide for the Brazilian Population, particularly through the predominance of fresh and minimally processed foods and the diversity of foods offered. Nevertheless, important opportunities for improvement were identified. Although animal-based foods represented less than one-third of total energy intake, they accounted for most of the carbon and water footprints associated with the analyzed menus and were the primary contributors to excess protein and saturated fat intake.

The findings highlight a clear imbalance between the nutritional and environmental dimensions of current school menus, suggesting that sustainability objectives embedded in national policies have not yet been fully translated into practice. In this context, the simulated strict vegetarian menu demonstrated that substantial reductions in greenhouse gas emissions and water use can be achieved while maintaining compliance with nutritional recommendations. These results reinforce growing international evidence that greater reliance on plant-based foods can simultaneously support human health and environmental sustainability.

Beyond its nutritional function, school feeding is a strategic public policy instrument that can shape healthier, more sustainable food systems. Given the scale and reach of the Brazilian school feeding program, incorporating environmental criteria into menu planning and food procurement could generate significant public health and ecological benefits. The proposed intervention offers a practical example of how sustainability goals may be integrated into school feeding without compromising dietary quality.

Future studies should evaluate the economic feasibility, operational implementation, acceptability, and long-term impacts of plant-based school meal strategies among students, families, and school communities. Such evidence will be essential for developing school feeding policies that effectively address the interconnected challenges of nutrition, food security, and environmental sustainability.

## Figures and Tables

**Table 1:** Nutritional profile of the sample of 130 menus analyzed and PNAE recommendations.

Nutrients	Mean (SD)	Percentage of TEI	Recommended Values
Carbohydrate	175.4 g (27.9)	58.8%	55% to 65% TEI
Protein	57.6 g (12.8)	19.3%	10% to 15% TEI
Total fat	29.0 g (6.2)	21.9%	15% to 30% TEI
Fiber ^1^	13.0 g (6.4)	-	-
Saturated fat	11.6 g (6.3)	8.8%	Up to 7% TEI
Sodium ^2^	797.5 mg (183.9)	-	Up to 1400 mg
Added sugar	15.9 g (9.3)	5.3%	Up to 7% TEI
Calories ^1^	1190.3 kcal (128.2)	-	-

TEI: Total energy intake; SD: Standard deviation; PNAE: National School Feeding Program. Source: Resolução Nº 6/2020 (Resolution No. 6/2020). ^1^ The PNAE does not have explicit recommendations for fiber and calories. ^2^ Sodium recommendations are expressed in mg and not as a percentage of TEI.

**Table 2:** Suggestion of a strict vegetarian menu featuring new foods.

	Groups Distribution	Proposed Foods
	Fruit group	Papaya
	Grain group	Couscous
Meal 01	Bean (legume) group	Tofu
	Culinary ingredients	Salt
	Culinary ingredients (3 pc)	Soybean oil
	Vegetable group	Lettuce
	Vegetable group	Tomato
	Grain group (1/2 pc)	Rice
Meal 02	Roots and tubers group (1/2 pc)	Potato
	Bean (legume) group	Black bean
	Bean (legume) group	Chickpeas
	Culinary ingredients	Salt
	Culinary ingredients (3 pc)	Soybean oil
	Vegetable group	Carrot
	Vegetable group	Tomato paste
	Grain group	Pasta
Meal 03	Bean (legume) group	Lentils
	Bean (legume) group	Textured soy protein
	Culinary ingredients	Salt
	Culinary ingredients (3 pc)	Soybean oil

pc: Per capita.

**Table 3:** Nutritional profile of the proposed vegetarian menu featuring new foods.

	Recommended Values	Values Obtained
Saturated fat	Up to 7% TEI	3.3%
Total fat	15% to 30% TEI	25.8%
Carbohydrate	55% to 65% TEI	61.7%
Protein	10% to 15% TEI	12.4%

TEI: Total energy intake.

## Data Availability

The original data presented in the study are openly available in FigShare at https://figshare.com/s/7a069d57a84cd6c8d6d8, accessed on 29 June 2026.

## Abbreviations

The following abbreviations are used in this manuscript:

PNAE	National School Feeding Program
TACO	Brazilian Food Composition Table
TBCA	Brazilian Food Composition Table of the University of São Paulo
TEI	Total Energy Intake
SVB	Brazilian Vegetarian Society
